# Deep Flexible Sequential (DFS) Model for Air Pollution Forecasting

**DOI:** 10.1038/s41598-020-60102-6

**Published:** 2020-02-25

**Authors:** Kıymet Kaya, Şule Gündüz Öğüdücü

**Affiliations:** Istanbul Technical University, Department of Computer Engineering & ITU AI Research and Application Center, Istanbul, 34467 Turkey

**Keywords:** Environmental sciences, Engineering, Mathematics and computing

## Abstract

Growing metropolitan areas bring rapid urbanization and air pollution problems. As diseases and mortality rates increase because of the air pollution problem, it becomes a necessity to estimate the air pollution density and inform the public to protect the health. Air pollution problem displays contextual characteristics such as meteorological conditions, industrial and technological developments, traffic problem etc. that change from country to country and also from city to city. In this study, we determined PM${}_{10}$ as the target pollutant and designed a new deep learning based air quality forecasting model, namely DFS (Deep Flexible Sequential). Our study uses real world hourly data from Istanbul, Turkey between 2014 and 2018 to forecast the air pollution 4, 12, and 24 hours before. DFS model is a hybrid & flexible deep model including Long Short Term Memory (LSTM) and Convolutional Neural Network (CNN). The proposed model also is capable of generalization with standard and flexible Dropout layers. Through flexible Dropout layer, the model also obtains flexibility to adapt changing window sizes in sequential modelling. Moreover, this model can be applied to other air pollution time series data problems with small modifications on parameters by taking into account the nature of the data set.

## Introduction

Air pollution plays an important role in living conditions in most large cities of the world. Accurate estimation of air pollution is a preliminary step in the presence of air pollution control technologies and helps to ensure economic and social development in developing countries.

There are standard approaches in order to identify specific pollutant mixtures that may include hundreds of gas compounds and particulates of complex physic-chemical compounds. These mixtures which are combinations of different pollutants in varying percentages, depend on social, economic, and technological activities at a given area. So, in air pollution studies, air pollutant indicators are used for risk assessment and epidemiological analysis. Most common indicators are particulate matter under $10\ \mu m$ (PM${}_{10}$), particulate matter under $2.5$  $\mu m$ (PM${}_{2.5}$), nitrogen oxides (NO, NO${}_{2}$, NO${}_{X}$), ozone (O${}_{3}$), sulphur oxides (SO${}_{2}$), and carbon oxides (CO).

Air pollution has serious effects on urban residents, especially vulnerable ones such as children and people with heart or respiratory failure. Besides, growing mortality and morbidity rates are associated with the high density of pollutants in the air (e.g. PM and SO${}_{2}$)^1–3^.

Particulate matters are among air pollutants with serious effects on human health. Both heavy metals and carcinogenic chemicals such as mercury, lead, and cadmium lead serious health problems. Gasoline and diesel powered vehicles emit particulates such as benzo(a)pyre- ne and cause cancer when inhaled for a long time^4^. Prolonged exposure to high concentrations of PM${}_{10}$ may also lead to early deaths, impaired cardiovascular system, internal diseases and respiratory infections. Considering the threats posed by human health, in this study we focus on estimating PM${}_{10}$ density.

Estimation of alterations at air pollution concentration is required to secure life quality at city centers. In this respect, air quality estimation models have been developed in order to forecast air pollution before air quality declines significantly at the regional or local level. While doing this, the characteristics of atmospheric pollution and their negative effects on life quality are taken into account^5,6^.

In previous studies, meteorological data are widely used to forecast/predict air quality. Meteorological conditions play a pivotal role in determining air pollutant concentrations^4,7–11^. For instance, subnormal temperatures and solar radiation slow down photo-chemical reactions and lead to low levels of secondary air pollutants such as  O${}_{3}$^ 10^. Increasing wind velocity may either increase or decrease air pollutant concentration^12^. High wind velocity can lead to dust-storms by levitating particulate matter from the surface^13^. A high level of humidity generally increases concentration of PM, CO, and SO${}_{2}$ in the air. Meanwhile it may decrease the concentration of some pollutants such as NO${}_{2}$ and  O${}_{3}$^12^. This is because high humidity is an indicator of rain^14^.

Besides meteorological data, pollution data can also be used for air quality forecasting. Nevertheless use of pollution data is rare than meteorological data due to three obstacles. First, the establishment and administration of an air quality monitoring station (AQMS) is more costly and difficult than that of meteorological station. Second, AQMSs are founded at very rare and specific locations. Finally, data collection from AQMSs is difficult.

This study aims to forecast PM${}_{10}$ density four, twelve and twenty-four hours before it occurs and offers a novel deep learning based forecasting approach, entitled Deep Flexible Sequential (DFS) model. The novelty of our model lies in the combination of an Convolutional Neural Network (CNN) and Long Short Term Memory (LSTM) which yields a flexible dropout layer. The model we propose here, is a hybrid-sequential model that incorporates a Convolutional 1D (Conv) layer and a LSTM layer and combines their advantages. Thanks to Conv, feature extraction is effectively performed and with the help of LSTM, long-short term time dependencies are taken into account sequentially. Outside the Conv and LSTM layers, dropout layers are included in the model in order to prevent overfitting.

For the purpose of observing our DFS model performance in air pollution forecasting, we gathered both hourly meteorological and pollution data between August 2014 and February 2018 from four stations located in a very central location in Istanbul, Turkey. Additionally, we also collected traffic data (https://uym.ibb.gov.tr/) that we think it has a great impact on air pollution in urban areas. Proposed DFS architecture uses past meteorology, pollution, traffic and PM${}_{10}$ data and implements deep learning based hybrid-sequential modeling for future PM${}_{10}$ forecasting.

We compared the success of DFS model with deep learning-based Gated Recurrent Unit (GRU), LSTM, bidirectional LSTM (bi-LSTM) and Conv-LSTM models through the MAE and RMSE metrics for three different window sizes (g = 4, 12, 24) in four different measurement stations. The experiments demonstrate that proposed DFS model architecture is more suitable than the state of the art deep learning methods.

The contributions of this study are as follows:

- We developed a new flexible and hybrid deep learning model called DFS for future PM${}_{10}$ forecasting. Our model has generalization ability on different regions and includes CNN, LSTM and Dropout layers together. Compared to pure deep learning models, the hybrid architecture combining the benefits of these layers clearly comes to fore.
- DFS air pollution forecasting model uses multivariate time-series data related to air pollution and performs flexible-temporal modeling regardless of window size. DFS can be an inspiration to not only other air pollution forecasting studies but also different data mining problems that perform sequential modeling on time series data with the flexibility it provides.
- The fact that obtaining meteorological data is relatively easy compared to pollution data makes the estimation models using meteorological data more easily applicable. However, pollution data, including other pollutants at the point of measurement outside the target pollutant, may contribute more to the estimation. Our model has produced satisfactory results on two different data sets and by adding traffic data to these data sets, inter-data interaction from different sources is provided.

The rest of the paper is organized as follows. Previous air quality-pollution forecasting studies in literature are described in *Related Works*; proposed DFS model and the deep learning methods in the background of the model are described in *Methodology*. Subsequently, *Model Implementation and Experimental Results* section includes data analysis, data preprocessing, step-by-step formation of the DFS model and the experimental results. Lastly, *Conclusion and Future Work* concludes the paper.

## Related Works

The approaches to estimate PM${}_{10}$ density in the air can be categorized into two major groups based on the techniques they applied: deterministic models and statistical models. Deterministic models are methods that quantify the deterministic relationship between emission sources, meteorological processes, physco-chemical changes and pollutant concentrations, including the consequences of past and future scenarios and the determination of the effectiveness of alleviation strategies. On the other hand, statistical models include linear and nonlinear supervised learning methods and are easily distinguished from deterministic methods by their randomness property.

Machine learning approaches from statistical models proved their superiority to deterministic models in many air pollution estimation studies.

### Studies with target pollutants other than PM

Singh et al. predicted SO${}_{2}$ and NO${}_{2}$ by using meteorological parameters^15^. In their work, they compared linear (Partial Least Square Regression (PLSR)) and non-linear models (Multivariate Polynomial Regression (MPR), Artificial Neural Networks (ANN)) and found most accurate results with ANN model.

Among different ANN approaches (Multilayer Perceptron Network (MLPR), Radial-basis function network (RBFN), Generalized Regression Neural Network (GRNN)), GRNN outperformed others. Ana Russo and colleagues highlighted the importance of size reduction^16^ and predicted NO${}_{2}$, NO and CO densities with meteorological parameters such as temperature, relative humidity, precipitation accumulation, atmospheric boundary layer height, pressure, and brightness. Dhirendra Mishra^17^ compared Multiple Linear Regression (MLR) and Principle Component Analysis (PCA) aided ANN model while forecasting hourly NO${}_{2}$ concentration in Tac Mahal, India. Because the latter model displayed a better performance it has been stated that the model can be used for air pollution forecasts in Tac Mahal, Agra. In another study Multilayer Perceptron (MLP) is used to forecast the concentrations of NO${}_{2}$, O${}_{3}$, and SO${}_{2}$ in Delhi, the second biggest city in India^18^.

Sheikh Saeed Ahmad and his colleagues emphasized feature engineering and predicted the NO${}_{2}$ density at Rawalpindi and Islamabad regions between November 2009 and March 2011 via temperature, relative humidity, precipitation accumulation, the location on earth, the week of measurement, and the location number^19^. The location number relied on the sequential binary number system. The number became ‘1’ if bidirectional transport way, main road, side road, public hospital, modern residence, trading area, resting area, bus station, school, lake, or, forest exists nearby the area. If there was not any of these, then it was coded as 0. The best ANN network structure was decided by evolutionary algorithm and the results were improved by back-propagation method.

### Studies where PM is the target pollutant

Haiming *et al*. used PM${}_{10}$, SO${}_{2}$, NO${}_{2}$, temperature, pressure, wind direction, wind velocity as parameters while predicting PM${}_{2.5}$ concentration in^20^. It was understood that RBF with Gauss transfer function generated more accurate result than ANN with back-propagation method. Similarly^21^, used ANN in order to forecast PM${}_{10}$ density in Barcelona and Montseny. Nieto followed a similar method^22^ in Ovieodo utilizing monthly data. In 2011, Mingjian and colleagues employed PM${}_{2.5}$, PM${}_{5}$ and PM${}_{10}$ density data collected from laser dust monitors located along the Zhongshan Avenue, which is one of the most busy streets in the city of Chongqing in China^23^. While predicting PM${}_{2.5}$ density^24^, utilized both the Aerosol Optical Depth (AOD) provided by satellite images and the traffic density. On the other hand, MODerate resolution Imaging Spectro-radiometer (MODIS) used the average of satellite-based night lights in addition to AOD satellite images while estimating PM${}_{10}$^25^. In their recent study, Kurt and Oktay built a geographic model while forecasting SO${}_{2}$, CO and PM${}_{10}$ levels at Beşiktaş region in Istanbul by using daily air pollutant data, meteorological data, and geographic data^26^.

In air pollution estimation studies via machine learning, it is clearly seen that methods based on artificial neural networks stand out regardless of whether the target pollutant is PM or not. Considering the success of deep learning techniques in many other application domains^27–29^, it is inevitable that the studies for air pollution prediction have recently focused on deep learning methods.

First studies conducted with deep learning in this area have tended to use pure sequential models (RNN, LSTM, GRU) with proven success in time series. A cyclic ANN model, Recurrent Neural Network (RNN), was run for estimating the density of PM${}_{10}$ and PM${}_{2.5}$ in the work of Kim and his colleagues^30^. RNN performance was compared with Feed Forward Artificial Neural Network (FFANN) and MLR on the data from subway stations in Seoul, the capital city of Korea. The findings of this study demonstrated that compounds with Nitrogen element are more effective at predicting PM${}_{10}$ and PM${}_{2.5}$ than the compounds with Carbon element. Comparing RNN, RNN based-LSTM and RNN based-GRU performances, GRU was found to be slightly higher than LSTM for PM${}_{10}$ level prediction^31^. The extended version of LSTM is presented as framework in^32^ by using hourly PM data.

Convolutional neural networks, which stand out with its success in image processing, are used in many research areas for feature extraction. In air pollution estimation problems Conv takes place in hybrid network architectures with sequential models in general. Study of air pollution prediction through ozone in^33^ and PM${}_{2.5}$ forecasting studies in^34,35^ are some of these hybrid models.

Air pollution is present in every scale from personal to global. The outcomes of ambient air pollution may be divided into two as local outcomes and global outcomes. While local outcomes have an impact on human health, vegetation, raw material and cultural goods, global outcomes may cause greenhouse effect, climate change and tropospheric/stratospheric ozone effect.

In this study, air pollution in Istanbul,Turkey is predicted accurately four, twelve and twenty-four hours before air pollution occurs using deep flexible sequential model, namely DFS. This is a hybrid deep learning model including LSTM, Conv and Dropout layers. The novelty in this model is the use of flexible dropout layer, which distinguishes our DFS forecasting model from other air pollution forecasting studies using hybrid deep learning methods. On the other hand, crucial difference between this work and the previous sequential modelling works is that we emphasize how flexible deep model should be designed on time series data due to changing window sizes. The proposed DFS model has a different architecture than the models proposed so far and can be used in other air pollution forecasting studies in the future.

## Methodology

The densities of pollutants are influenced by meteorological parameters, which display specific characteristics on a hourly, daily, yearly basis. That is, not surprisingly, the highest air pollutant densities in Istanbul are measured not only in summer months due to high temperatures and evaporation but also in winter months due to high level of gasoline consumption. Therefore, we can say it is contrary to the nature of the problem to use fully connected artificial neural networks by treating the air pollution estimation problem independent from the time series feature. For such problems, sequential deep learning methods are already available in the literature.

Studies using traditional prediction methods view time as a feature and do not use previous target values at prediction model. However, pollutant density at time *‘t’* is influenced by the value at time *‘t-g’* as well. Traditional artificial neural networks do not forecast with sequential information so their connections with previous events are limited. At this point, RNNs come to fore. RNNs differ from traditional networks because they provide a continuity of information flow thanks to their cyclic nature.

This study uses RNN based GRU, LSTM, bi-LSTM, and hybrid model Conv-LSTM for performance evaluation. In the following sections; the basis of sequential modeling in deep learning, RNN, and our proposed DFS model architecture with its background methods are presented.

### RNN

RNNs have proven useful at time series^36^, natural language processing^37^, and bioinformatics^38,39^. In short, these ANNs yield satisfactory results at applications that use serial and connected data sets. While feeding the network, RNNs take and process input series at each stage. They hold these series at a hidden unit and use this information in order to update state vector that keeps information about all previous elements of the series.

**Figure 1:**
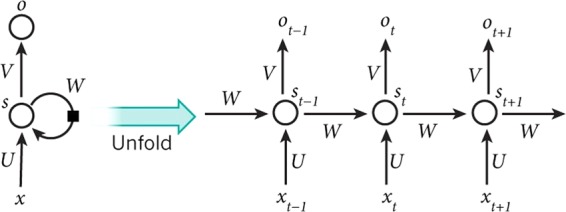
RNN Architecture^40^.

  Figure 1 shows both the architecture of the RNN and its unfold version. The symbols shown in the figure are as follows: x${}_{t}$ is input sequence, o${}_{t}$ is output vector, s${}_{t}$ is hidden state vector and W, U, V weight matrices. RNN maps an input sequence (x${}_{t}$) into an output sequence (o${}_{t}$) according to the recursive formulas of RNN in Eqs. 1 and 2.

1 $${s}_{t}=tanh(W{s}_{t-1}+U{x}_{t})$$

2 $${o}_{t}=V{s}_{t-1}$$

When looked at unfold version of the architecture (time is not cyclic) in Fig. 1, it is not wrong to state that RNN is a very deep version of FFANN where same weights are shared. However, the drawback of RNN is salient during network training, where multiplicative decreases/increases in back-propagated gradients lead to Vanishing Gradient^41^ or Exploding Gradient^42^ problem. When gradient problems occur, the training process takes too long and the accuracy is decreasing. Another problem regarding RNN is that although its primary objective is to learn long-term dependencies, it is not very good at storing network information especially when retrospective dependencies abound. In order to fix these, RNN-based LSTM model is suggested.

### DFS model for air pollution forecasting

**Figure 2:**
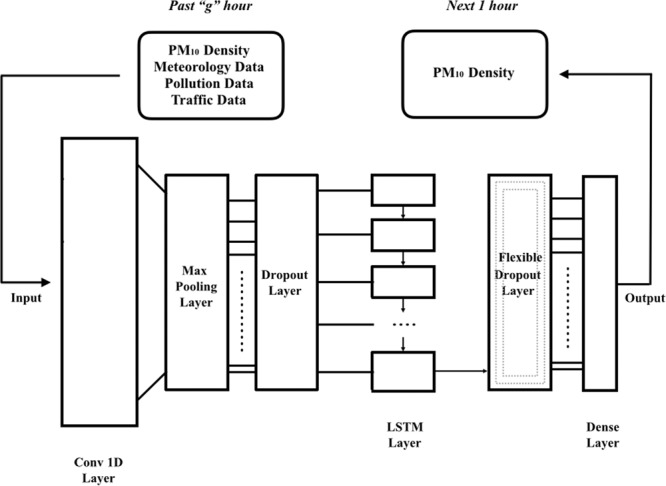
Deep Flexible Sequential Model for Air Pollution Forecasting.

This study proposes the Deep Flexible Sequential (DFS) model in Fig. 2 for air quality forecasting problem. The model includes LSTM and Convolutional layers and becomes prominent with its flexible Dropout layer. Before giving the details of the DFS Model architecture we propose, LSTM and CNN are described in the following subsections which form the basis of the proposed model.

#### LSTM

**Figure 3:**
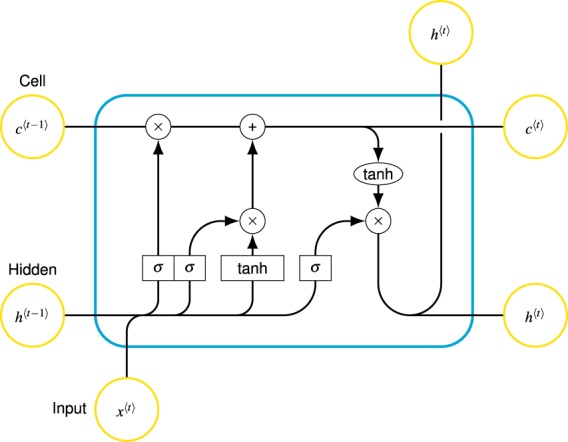
Long-Short Term Memory Architecture.

LSTM^43^ is a special version of RNN and is essentially separated from RNN by the fact that each neuron in its structure is actually a memory cell. As shown in Fig. 3, the working principle of LSTM relies on cells and intercellular data transfer. Information obtained from previous memory cells is used when processing in the current cell. In this wise, data is transferred from one cell to another and temporal dependencies are stored.

LSTM can handle even the longest sequence data without being affected by gradient problems and proves useful at learning long term dependency. Compared to similar methods, it performs better than GRU^44^ and RNN especially while modeling long distance relations and differs from other types of learning models with its three-gate structure (forget gate, input gate, output gate).

Long-Short Term Memory Architecture is presented in Fig. 3, where $x(t)$ is input of current cell, $C(t)$ is the cell memory, $h(t)$ is output of current cell block to be used in the next cell as a hidden state. $C(t-1)$ and $h(t-1)$ comes from previous cell and ensures sequential dependency. "$\sigma$” is $Sigmoid$ and "$tanh$” $HyperbolicTangent$ functions. While implementing element-wise weighted sum operation in LSTM, "$\times$” shows element-wise multiplication and "+” indicates element-wise sum.

- **Forget Gate:** At the forget gate, the decision is about how many percent of the information from previous cell is preserved in the new cell. The output from previous cell $h(t-1)$ is combined with the input of current cell $x(t)$ and this combination is introduced into the $Sigmoid$ function in Eq. 3. Afterwards, according to the multiplication of the output of $Sigmoid$ activation function and $C(t-1)$, it is decided to which extent the existing information is forgotten (Eq. 4). The output by $Sigmoid$ is between 0 and 1, where 0 denotes complete forgetting, whereas 1 does complete remembering.
  3 $$\quad S(t)=\frac{1}{1+{e}^{-t}}$$
  4 $${f}_{t}=\sigma ({W}_{f}\cdot [{h}_{t-1},{x}_{t}]+{b}_{f})$$
- **Input Gate:** This layer is composed of $Sigmoid$ layer and $tanh$ layer. The former decides which values will be updated (Eq. 5), whereas the later generates possible values of ${\widetilde{C}}_{t}$ vector (Eq. 6). The outputs of these two layers are multiplied by element-wise multiplication and the result is added to the function $C(t)$ as in Eq. 7.
  5 $$\quad {i}_{t}=\sigma ({W}_{i}\cdot [{h}_{t-1},{x}_{t}]+{b}_{i})$$
  6 $${\widetilde{C}}_{t}=tanh({W}_{C}\cdot [{h}_{t-1},{x}_{t}]+{b}_{C})$$
  7 $${C}_{t}={f}_{t}\ \ast \ {C}_{t-1}+{i}_{t}\ \ast \ {\widetilde{C}}_{t}$$
- **Output Gate:** This layer decides cell state output at time ‘t’. Then, the output of h(t-1) and the input of X(t) are combined and the result is put into $Sigmoid$ function (Eq. 8). The output of this function determines how much information will be retrieved from cell state. $C(t)$ results of Forget gate and Input gate are activated by $tanh$ function, and afterwards these results are multiplied by $Sigmoid$ output in order to yield the cell output (Eq. 9).
  8 $${o}_{t}=\sigma ({W}_{o}\cdot [{h}_{t-1},{x}_{t}]+{b}_{o})$$
  9 $${h}_{t}={o}_{t}\ \ast \ tanh({C}_{t})$$

#### CNN

CNN is more prominent in image processing^45^ and computer vision^46^ than that in most of the deep learning studies, CNN and image are mentioned together. Yann Lecun’s LeNet-5, AlexNet, GoogLeNet and VGG are the keystones of studies using convolutional networks in image processing. These are followed by modern network architectures such as Inception, ResNet and ResNeXt.

Although CNN is particularly well known for its success in visual imagery analysis, it can also be effectively applied to time series analysis problems. What makes CNN different from other networks is basically weight sharing and sparse connectivity. Through the shared weights, training is relatively easy on CNN compared to FFANN.

The weight sharing and local perception features make CNN attractive for time series models as it reduces the number of parameters and improves the learning ability of the model. Hierarchical CNN structures for feature extraction consist of two successive layers; first convolutional layer and then subsampling or pooling layer. In the DFS architecture, 1-dimensional CNN (CNN-1D) is used for property extraction, and CNN-1D here contains the maximum pooling layer after the convolution layer. The convolutional layer, implements sliding-window on input data and by this way, it creates feature maps that represent the temporal sequence property of time series data. The weight of the convolution filter is shared in the convolutional layer and connected to the input. The maximum pooling layer reduces the size of output dimension over the feature maps in the convolutional layer. Therefore, it may improve the learning and generalization ability of the model by ignoring temporal shifts and distortions in data.

## Model Implementation and Experimental Results

In this study, air quality intensity is predicted before air pollution occurs by using hourly data at $(t-4)$, $(t-12)$, and $(t-24)$. Real world data used in here, belong to Aksaray, Alibeyköy, Beşiktaş and Esenler which are located in a very central location in European side of Istanbul covering the time period between August 2014 and February 2018.

Meteorological conditions play a critical role while measuring air pollutant concentration. Therefore, temperature in ${}^{\circ }$C (maximum temperature, minimum temperature), wind speed, wind direction, maximum wind speed, maximum wind direction, and humidity meteorological parameters were collected on hourly basis from the Turkish State Meteorological Service (TSMS). For reliable temperature and wind values, a number of serial measurements is carried out within the same hour. Meteorological parameters with max-prefix denote the highest value of a given parameter within an hour.

As for air quality prediction, it is found that other pollutants can be used for measuring the density of target pollutants^47^. The pollution density data of CO, NO, NO${}_{2}$, NO${}_{X}$, O${}_{3}$ and SO${}_{2}$ were collected from the closest AQMSs to meteorological stations.

Meteorological and pollutant data, which are frequently used at air pollution prediction/forecasting, yield satisfactory results in many studies. Nevertheless, works in^48^ and^49^ suggest to add traffic data into data sets in further studies. Both of these studies solve air pollution problem by times series prediction and specifically uses LSTM. Given that Istanbul is a crowded mega city and suffers from traffic, we believed that taking traffic data into account would increase the performance of our model. For this reason, traffic data from Istanbul Metropolitan Municipality are also included while predicting PM${}_{10}$. Traffic data is the percentage of traffic density measured with five-minute intervals (traffic index). In order to convert these five-minute data to hourly basis, we calculated their arithmetic mean. Since the singularity of these data may produce misleading forecasts, traffic data were used together with meteorological or pollution data. Air pollutant density values should be positive (http://havaizleme.gov.tr/). The hours at which the pollutant density is zero or negative is equivalent to no measurement at that time. Zero, negative values, or the lack of measurement at a given hour would create the sparsity problem. Since our missing data is negligible, we preferred to remove these samples from the data set instead of applying one of the data filling methods. After removal of the samples with missing data, we proceeded with the data at hand of which minimum, maximum, mean and standard deviation of PM${}_{10}$ values and number of samples in each data set are shown at Table 1.

**Table 1:** Descriptive Properties of PM${}_{10}$ for Regions.

	*PM*${}_{{\bf{10}}}$[${\boldsymbol{\mu }}{\boldsymbol{g}}$/${{\boldsymbol{m}}}^{{\bf{3}}}$]				
	Minimum	Maximum	Mean	Standard Deviation	Number of Samples
Aksaray	0.000023	982.71	63.92	47.9079	32128
Alibeyköy	0.000002	888.40	50.75	46.0379	39993
Beşiktaş	0.016733	970.14	46.07	36.6414	38992
Esenler	0.004611	957.75	56.59	44.5459	49068

In meteorological data set, wind direction and maximum wind direction parameters are represented by a value between 0 and 360. These features differ from others as they are categorical variables. Expression of these categorical features by 4, 8 and 16 labeling was applied and tested. For instance, in labeling 4; 0–90, 90–180, 180–270, and 270–360 intervals are represented by 1, 2, 3, and 4 respectively. After feature representations were changed, *’One Hot Encoding’* was applied and the effects of these representations on prediction models were compared. It is understood that 4-label representation gave better results than other labels or unmodified representation. So, 4-label representation was used in our models.

In order to evaluate the performance of the model, state of the art techniques RNN based LSTM, GRU, bi-LSTM and hybrid method Conv-LSTM used in this study implemented in Keras (supports Tensorflow backend) framework. In each region, data sets were divided into three as; training set 60%, validation set 15% and test set 25%. Thus, the three-year data was used for the training of the model, while the data of the last year was reserved for the test.

We built our model on LSTM in beginning, since the air pollution problem is based on time series data and developed this model step by step until the final DFS model was obtained. In recent studies at the field of air pollution forecasting, it is advocated that models with 1-2 LSTM layers outperform those with 3-4 layers^31^. Similarly, Chaudhary et al. showed that the model with single layer and 50 memory units yielded the best results^50^. So we thought that to use an initial model with single layer of average depth and LSTM with 96 memory units would be more appropriate. Since LSTM models give more accurate results on data at the interval of 0-1, we converted the data to 0-1 range by using MinMaxScaler^51^. This transformation also made computational time shorter.

During hyper-parameter tuning for the model of single layer LSTM with 72 memory units model, we used the meteorological data that performed best in our previous study^52^ and forecasted PM${}_{10}$ density at time t by using data we have at (t-4), (t- 12), and (t-24). We evaluated model performance that depends on parameter changes according to MAE (10) and RMSE (11). We also repeated the parameter optimization process mentioned for 48, 96, 120 and 144 LSTM unit values in stations Aksaray, Alibeyköy, Beşiktaş and Esenler.

10 $$MAE=\frac{1}{n}{\sum }_{1}^{n}\left|{e}_{t}\right|$$

11 $$RMSE=\sqrt{\frac{1}{n}{\sum }_{t=1}^{n}{e}_{t}^{2}}$$

We observed that LSTM Model with 96 units stands out in all regions. So, in order to demonstrate the effect of the parameters on the model, we present the results of the initial LSTM Model with 96 memory unit for Beşiktaş, which is relatively more lively area and prone to air pollution at Table 2.

**Table 2:** First Step of DFS Model - LSTM Model with 96 Memory Unit for “Beşiktaş”.

Window size (g) :				g = 4					g = 12					g = 24				
Optimizer	Loss Function	Batch Size	Performance Metric	50	80	100	150	200	50	80	100	150	200	50	80	100	150	200
Adam	mae	48	MAE	**7.90**	**7.82**	**7.79**	**7.93**	**8.13**	**8.06**	**7.85**	**8.09**	**8.39**	**8.47**	**7.65**	**7.69**	**7.69**	**8.40**	**8.04**
			RMSE	16.70	17.19	17.45	18.57	19.87	16.47	17.53	17.15	23.96	22.76	16.70	16.71	16.87	25.36	19.09
	mse		MAE	8.62	8.92	8.48	8.23	8.91	8.51	8.53	9.07	9.82	8.85	8.41	8.66	9.01	9.15	8.83
			RMSE	20.23	20.92	18.85	18.91	22.43	17.82	19.58	20.95	25.14	22.42	19.38	22.21	23.39	23.46	22.38
RMSProp	mae		MAE	11.79	9.08	9.56	10.33	10.38	15.01	14.18	12.28	9.41	10.86	8.87	9.63	8.80	9.60	11.05
			RMSE	19.54	18.98	22.92	23.45	25.70	21.48	22.73	22.41	24.38	21.41	17.45	16.20	18.02	19.65	24.31
	mse		MAE	13.99	14.50	14.73	14.70	8.99	11.82	13.71	12.49	8.52	9.48	12.70	8.80	13.48	13.67	14.88
			RMSE	22.17	22.85	24.83	22.05	23.52	19.60	20.30	21.10	18.13	20.17	25.94	25.74	24.88	25.62	26.25
Adam	mae	96	MAE	**7.50**	**7.58**	**7.64**	**8.27**	**14.04**	**8.16**	**7.92**	**8.74**	**9.42**	**13.66**	**7.46**	**7.30**	**7.53**	**8.20**	**8.83**
			RMSE	16.36	16.60	16.36	16.71	22.16	16.79	16.41	17.04	18.20	23.07	16.37	16.31	16.33	19.72	23.10
	mse		MAE	8.83	8.40	8.74	10.72	12.32	8.70	8.46	9.17	9.08	10.17	8.70	8.56	8.73	8.79	11.44
			RMSE	19.10	19.43	20.93	24.06	25.79	18.41	20.83	22.02	20.84	25.78	18.84	20.33	20.05	21.52	26.75
RMSProp	mae		MAE	9.13	8.92	8.90	9.13	8.71	16.03	14.92	13.56	9.08	9.81	7.87	7.87	8.65	8.56	8.03
			RMSE	17.23	17.35	18.23	23.73	19.05	22.11	22.62	23.35	20.75	23.29	16.86	18.38	21.21	24.18	19.36
	mse		MAE	11.88	11.28	9.52	8.73	9.18	14.72	13.07	14.89	10.38	8.20	8.39	9.36	8.43	11.19	9.30
			RMSE	20.54	19.46	17.99	22.19	21.24	23.40	21.32	24.51	19.91	20.19	21.64	22.79	25.37	30.75	21.56
Adam	mae	24	MAE	**7.83**	**7.89**	**8.01**	**8.31**	**8.10**	**7.92**	**7.77**	**8.01**	**8.09**	**8.68**	**7.71**	**7.95**	**8.15**	**8.25**	**8.53**
			RMSE	17.02	19.04	20.17	21.86	21.34	16.60	18.21	17.48	20.77	23.16	16.65	19.76	23.41	22.08	22.37
	mse		MAE	8.33	8.52	8.51	8.77	8.32	8.49	8.68	8.94	8.78	9.42	8.48	8.68	9.03	9.39	8.58
			RMSE	20.02	19.21	20.68	22.83	20.09	20.34	22.47	26.95	26.68	25.35	19.96	21.02	24.44	28.17	24.10
RMSProp	mae		MAE	9.87	9.24	10.33	12.15	13.28	10.79	10.77	10.29	11.85	8.39	11.00	9.94	12.95	11.19	9.60
			RMSE	18.55	20.34	25.27	24.78	30.70	18.87	20.21	20.13	21.51	20.50	19.86	17.58	22.08	24.06	23.39
	mse		MAE	12.26	13.30	12.99	15.92	11.29	15.42	14.63	14.96	15.37	16.46	12.08	15.95	14.45	12.45	11.49
			RMSE	20.35	20.53	22.57	25.62	22.95	26.38	24.52	25.87	24.63	30.08	26.63	26.56	29.46	21.67	24.06
Adam	mae	72	MAE	**7.72**	**7.59**	**7.56**	**7.76**	**8.05**	**8.93**	**8.24**	**8.01**	**8.54**	**8.48**	**7.45**	**7.56**	**7.53**	**7.91**	**8.48**
			RMSE	16.55	16.50	16.43	16.96	19.06	17.14	16.87	16.35	18.67	22.81	16.33	16.30	16.67	18.94	24.10
	mse		MAE	8.66	9.00	8.60	8.75	8.94	8.86	8.66	9.17	9.17	9.21	8.46	8.16	8.02	8.96	8.93
			RMSE	19.70	20.20	19.83	21.81	20.83	20.50	18.82	20.05	22.07	22.43	19.79	19.64	18.65	25.11	24.96
RMSProp	mae		MAE	8.85	8.87	9.10	8.99	8.96	15.69	14.07	12.14	13.64	11.69	8.88	8.68	8.58	8.42	8.50
			RMSE	17.35	19.50	19.99	18.20	18.86	21.89	21.19	20.67	25.28	21.47	18.16	17.37	23.11	23.27	21.81
	mse		MAE	11.30	10.10	8.44	8.50	8.39	13.66	8.40	14.39	14.13	13.95	10.92	8.18	8.58	8.25	8.35
			RMSE	20.34	18.84	18.61	19.46	20.99	20.91	17.32	21.19	22.6	22.46	22.93	23.61	28.92	22.30	26.33

As shown at Table 2, to use Adam^53^ optimizer rather than RMSProp optimizer yielded minimum error with less epoch values. After the selection of Adam optimizer, it is decided that the epoch value should be between 80 and 120. Accordingly, 100 was assigned as the epoch value since the *Training loss - epoch* graphic was saddle at that time.

When model performances were evaluated according to RMSE and MAE metrics, the former generated relatively greater results. It is so, because our target variable, PM${}_{10}$, takes values in a wide range (Table 1) and large values effect RMSE in proportion to their square. This is also valid for loss function. Unlike mse, mae does not take into account the square of the difference between the actual value and predicted value. On the other hand, loss function mse and performance metric RMSE yield results based on the total of these squares.

Batch size values are listed at Table 2 in order of progress. After output of models with 48 and 96 batch sizes are generated, 24 and 72 batch sizes were used respectively for hyper parameter optimization. Since we do time series forecasting on hourly basis data, we chose specifically 24 and multiples of 24 as batch sizes. The model with 72 batch size came to fore.

After the adjustment of the parameters as, optimizer = Adam, loss function = mae, batch size = 72, and epoch number = 100; we attained deep and shallow models by changing the memory unit values of LSTM. Alternative models to 72 memory unit model are those with 48, 96, 120, 144 memory units respectively. When we compared MAE values regarding test results based on these models (For instance, for g = 4 with different memory units the results are as follows; MAE${}_{48}$ = 7.69 MAE${}_{72}$ = 7.60, MAE${}_{96}$ = 7.56, MAE${}_{120}$ = 7.67, MAE${}_{144}$ = 7.75) the model with 96 memory units outperformed others.

**Figure 4:**
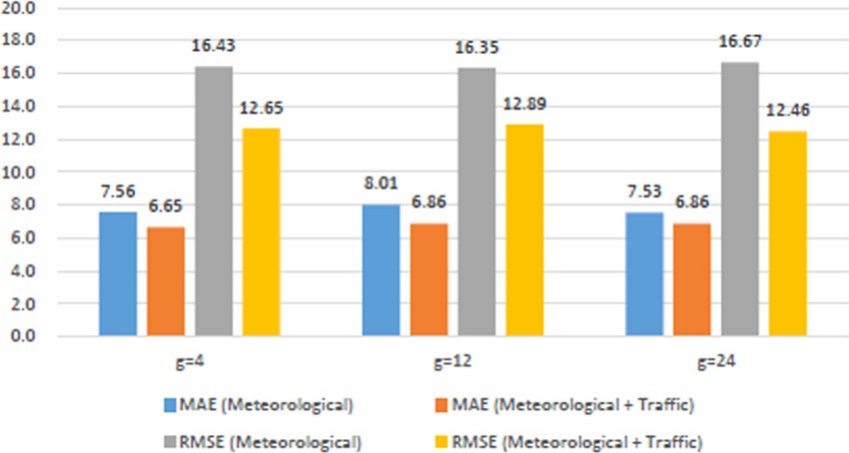
Meteorological Vs. (Meteorological + Traffic) for Beşiktaş.

**Figure 5:**
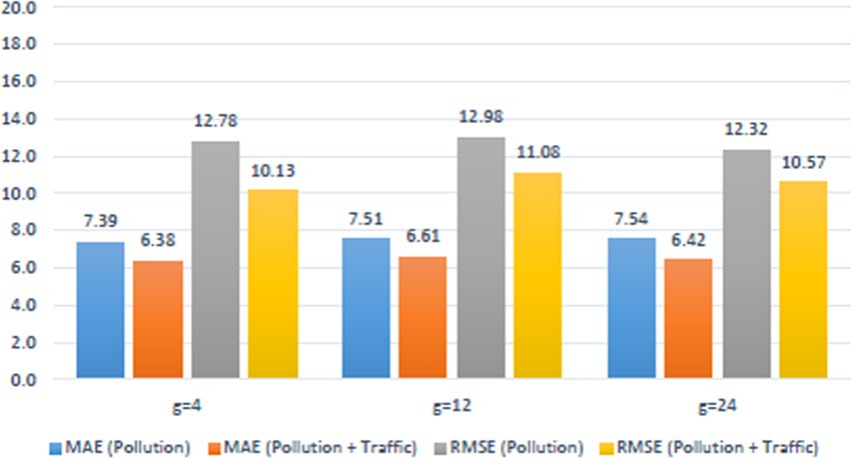
Pollution Vs. (Pollution + Traffic) for Beşiktaş.

By means of tuned LSTM model, we compared meteorological and pollutant data sets that included almost equal number of features and samples in order to see the effect of traffic data on Beşiktaş region. The results are illustrated as graphics at Fig. 4 for meteorological data and at Fig. 5 for air pollution data. Obviously, to include traffic data significantly minimizes errors.

We have trained our proposed Deep Flexible Sequential Model regarding two distinct data sets (meteorological and air pollution) each of which was added the traffic data. In DFS model, past sequential data is used as much as the window size. As shown in Eq. 12, sequence modeling was carried out in order to forecast the PM${}_{10}$ target value. The g value at the equation denotes window size we set as four, twelve and twenty four.

12 $$D(t-g),D(t-g+1),...,D(t-1),D(t)=D[{{\rm{PM}}}_{10}](t+1)$$

Numerous experiments with different hyper parameters have been conducted in order to construct the best deep neural network architecture. The best model after hyper parameter tuning is shown at Fig. 2 and DFS Model parameters are explained layer by layer below.

- Conv 1D Layer: Hyper parameters that we tuned in this layer are kernel size, number of filters and activation function. Also known as filter length, the kernel size set as 6 in this layer gives the size of the sliding window that convolves through the data. Filter defines how many sliding windows work on the data and also indicates how many features will be captured. Number of filters here is 24. Lastly, activation function is $tanh$.
- Max Pooling Layer: Max pooling was applied with ’pool size = 4’.
- Dropout Layer: In the dropout layer, the drop rate between zero and one is determined for the input (dropout rate = 0.2).
- LSTM Layer: This layer includes 24 LSTM memory units.
- **Flexible Dropout Layer**: This layer is based on the principle of defining Dropout Rate in time series problems as an equation that depends on window size. Thanks to this layer, flexible dropout rates can be assigned within a specific interval for each different window sizes in LSTM. This rate assignment is carried out by a multiplier value, which depends on a threshold value and window size ($g$).
  13 $$dropoutrate=0.19+0.0025\ \ast \ g$$
  In our study, window size ($g$) takes the values of 4, 12, and 24 and flexible dropout rate varies between 0.2 and 0.25 depending on the formula at Eq. 13.
- Dense Layer: Default parameters are used without any modification.

When we first designed our model, we used 0.2 dropout rate for both of two dropout layers and applied hyper parameter tuning for other layers. We made additional tests with lower and higher dropout rates in order to see whether the results would be better. Lower rates yielded no better results on any window size at the second layer. However, we observed less error values when higher dropout rates until around 0.25 are used especially for window sizes of 12 and 24. As for window size 4, increasing the rate from 0.2 to 0.25 decreased errors but beyond this rate the error values inflated. At this point, we concluded to design flexible dropout layer that depends on window size.

We applied different versions of flexible design to both dropout layers. The flexible design of the first dropout or both dropout layers increased errors. Therefore, we argue that the flexible dropout layer should be used after the LSTM rather than using before. By doing so, the error values of our model decrease. While using past sequential data, the bigger the window size the more features network can benefit from. As window size gets bigger, weight matrix grows and becomes complicate. We achieved to control this complexity by using Flexible Dropout Layer after LSTM layer.

Our model, LSTM, GRU, bi-LSTM and Conv-LSTM were applied to data sets, the model performances were compared, and MAE and RMSE error values are shown at Tables 3, 4, 5 and 6, respectively. One of the models compared here, bi-LSTM, has become very popular recently for its exemplary performance at natural language processing and machine translation. GRU occupies an important position in recommendation systems. When MAE and RMSE results are examined, it is found that our model led to a remarkable increase at the performance specifically for big window sizes. As for the other models, they can be ranked as LSTM, conv-LSTM, bi-LSTM, and GRU in terms of their performances. When the meteorological and pollution data sets are compared, it is seen that both data sets are sufficient for the proposed model since they revealed similar error values.

**Table 3:** Experimental Results in terms of MAE & RMSE for "Aksaray”.

Data Set	Window Size	*GRU*	*LSTM*	*bi-LSTM*	*conv-LSTM*	DFS	*GRU*	*LSTM*	*bi-LSTM*	*conv-LSTM*	DFS
		MAE					RMSE				
Meteorological + Traffic	**g = 4**	5.85	4.84	5.01	5.20	**4.32**	8.31	6.91	6.98	7.04	**6.24**
	**g = 12**	5.90	5.29	5.33	5.35	**4.45**	7.79	7.44	7.47	7.59	**6.49**
	**g = 24**	6.04	5.35	5.45	5.41	**4.45**	8.19	7.31	7.49	7.44	**6.49**
Pollution + Traffic	**g = 4**	9.02	7.87	8.51	8.12	**7.04**	14.93	13.33	13.99	13.61	**12.96**
	**g = 12**	8.63	7.20	8.20	7.68	**7.14**	14.36	14.08	14.16	14.22	**13.06**
	**g = 24**	9.38	8.18	8.52	8.16	**7.27**	16.10	14.48	14.54	14.44	**13.67**

**Table 4:** Experimental Results in terms of MAE & RMSE for "Alibeyköy”.

Data Set	Window Size	*GRU*	*LSTM*	*bi-LSTM*	*conv-LSTM*	DFS	*GRU*	*LSTM*	*bi-LSTM*	*conv-LSTM*	DFS
		MAE					RMSE				
Meteorological + Traffic	**g = 4**	7.90	6.53	9.97	7.20	**6.25**	15.56	15.23	16.68	15.33	**15.09**
	**g = 12**	8.54	8.53	9.61	9.02	**6.55**	15.44	15.34	16.29	15.81	**14.86**
	**g = 24**	7.76	6.64	9.44	7.78	**6.30**	15.16	14.37	16.35	14.89	**14.21**
Pollution + Traffic	**g = 4**	6.24	6.22	6.66	6.24	**6.00**	13.34	13.31	13.45	13.32	**13.15**
	**g = 12**	6.78	6.26	7.08	6.80	**6.08**	13.86	13.55	14.11	13.95	**13.41**
	**g=24**	6.55	6.29	6.87	6.69	**5.96**	13.65	13.44	14.20	13.91	**13.31**

**Table 5:** Experimental Results in terms of MAE & RMSE for "Beşiktaş”.

Data Set	Window Size	GRU	*LSTM*	*bi-LSTM*	*conv-LSTM*	DFS	*GRU*	*LSTM*	*bi-LSTM*	*conv-LSTM*	**DFS**
		MAE					RMSE				
Meteorological + Traffic	**g = 4**	8.10	6.64	6.65	6.68	**6.52**	14.06	12.12	12.65	12.73	**11.75**
	**g = 12**	8.12	6.85	6.86	6.90	**6.44**	13.99	12.74	12.89	13.04	**11.40**
	**g = 24**	9.10	6.80	6.86	7.02	**6.75**	15.01	12.46	12.49	13.56	**11.66**
Pollution + Traffic	**g = 4**	6.51	6.38	6.40	6.40	**6.29**	13.36	12.78	12.84	12.86	**11.47**
	**g = 12**	6.67	6.61	6.59	6.65	**6.48**	13.12	12.98	13.01	13.07	**12.23**
	**g = 24**	6.75	6.42	6.40	6.73	**6.35**	12.56	12.30	12.32	12.47	**12.21**

**Table 6:** Experimental Results in terms of MAE & RMSE for “Esenler”.

Data Set	*Window Size*	*GRU*	*LSTM*	*bi-LSTM*	*conv-LSTM*	DFS	*GRU*	*LSTM*	*bi-LSTM*	*conv-LSTM*	DFS
		MAE					RMSE				
Meteorological + Traffic	**g = 4**	7.70	7.63	7.85	7.68	**7.59**	11.94	11.85	12.11	11.90	**11.83**
	**g = 12**	7.74	7.56	8.25	7.70	**7.46**	11.83	11.66	12.71	11.72	**11.58**
	**g = 24**	7.74	7.58	7.79	7.68	**7.52**	11.84	11.67	12.03	11.68	**11.65**
Pollution + Traffic	**g = 4**	7.47	7.44	7.52	7.48	**7.41**	11.28	11.31	11.53	11.30	**11.32**
	**g = 12**	7.40	7.39	7.52	7.40	**7.32**	11.42	11.19	11.51	11.42	**11.16**
	**g = 24**	7.45	7.40	7.52	7.40	**7.29**	11.41	11.11	11.49	11.18	**11.09**

## Conclusion and Future Work

In this study, Deep Flexible Sequential Model is suggested since it yielded accurate predictions 4, 12 and 24 hours before the air pollution occurs. We are proposing a flexible deep learning model composed of CNN, LSTM, and Dropout layer. The contributions of these three components are as follows. First, CNN can reveal effectively the characteristics of the data. Second, LSTM shows a good performance while unfolding long time dependencies from time series data. Third, Dropout layer brings a balance during sequential modeling.

In this study, the performance of our model was compared with those of GRU, LSTM and bi-LSTM that prioritize sequential data at PM${}_{10}$ pollutant prediction. According to two performance metrics, MAE and RMSE, it is demonstrated that DFS Model displayed a superior performance. Under same parameters, DFS Model performed also better than Conv-LSTM model without flexible dropout layer structure (especially more salient at bigger window sizes) while predicting air pollution.

DFS model, which yielded remarkable results with four-year-long hourly data and eight features, is elaborately explained so that it can be used for air pollution forecasting at different regions. We believe that this model can also be used at different applications. We have two goals for future work: our specific goal is to collect data from other measurement stations in Istanbul and make a model fusing whole data, whereas our broad goal is to monitor the performance of DFS Model on different time series data sets beyond the air pollution problem.
